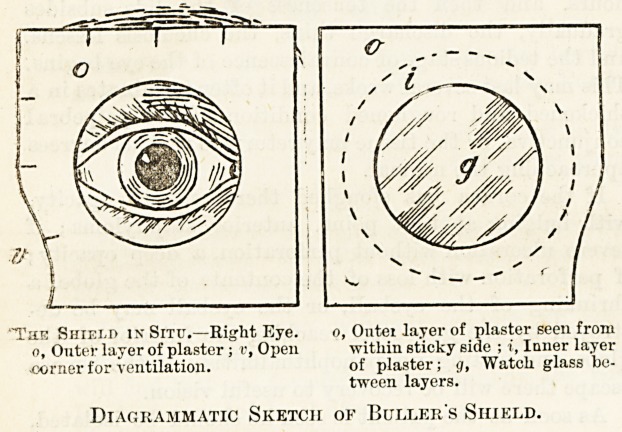# Gonorrhœal Ophthalmia

**Published:** 1900-01-13

**Authors:** Clements Hailes

**Affiliations:** Assistant Surgeon, Bristol Eye Hospital.


### Hospital Clinics and Medical Progress.

### GONORRHŒAL OPHTHALMIA.

By Clements Hailes, M.D., F.R.C.S.Edin., Assistant Surgeon, Bristol Eye Hospital.

Gonorrhœal ophthalmia is an acute purulent conjunctivitis characterised by the presence of the gonococcus in the discharge from the conjunctival sac. It is produced by direct inoculation of the conjunctiva by pus containing gonococci, is most severe and destructive, and a complaint to be greatly dreaded. Considering the general carelessness and often ignorance of those suffering from gonorrhœal urethritis, it is little short of a miracle that the ophthalmic affection is so comparatively rare, for when the pus is once conveyed to a healthy eye the gonococcus recognises a very suitable soil in which to cultivate its species, and a material in which it will thrive, and have but little resistance offered to its encroachments.

It may be conveyed straight from the urethra of an individual by means of his finger to his eye, or by using the towels, sheets, or other articles used by an infected person, or may be received from the eye of a person so infected by the nurse or medical attendant while dressing a case, especially where a syringe is carelessly used and causes some of the discharge to fly upwards, and, therefore, every care should be taken by persons attending to such cases. They should wear spectacles, not necessarily of any dioptric power, but as a shield when attending to the cases; should not put the face nearer the patient's than is absolutely necessary; should resist rubbing their eyes with their fingers until they have thoroughly cleansed and disinfected their hands by washing in a disinfectant solution; and should handle the patient's eye as little as possible with the unprotected finger. After its inoculation there is a brief period one to three days of (*a*) incubation, during which time there are no symptoms; this is followed by a stage of (*b*) invasion, commencing with irritation of the conjunctiva, a feeling of heat or pricking in the eyeball, which becomes injected, lachrymation and photophobia follow, the eyelids begin to swell, and a thin discharge to escape from the palpebral fissure. This stage is generally fully in force by the time the patient seeks medical advice, and the symptoms may be mild, or there may be exaggerated swelling, especially of the upper lid which may overlap the lower one, and be tight, hard, smooth, shining, and congested-looking, with pus oozing out beneath it, and hanging in drops from it. It may be impossible to raise the lid with the finger or retractor to get a view of the conjunctiva and cornea below it, in which case it is best to slit the outer canthus by inserting one blade of a probe-pointed scissors into the palpebral fissure, and pushing it as far as it will go in the outer canthus, and then cutting through; this not only enables one to manipulate the upper lid more easily, but by blood-letting lessens the congested state of the eye-lid. Now is the stage of (*c*) advance, during which the inflammation increases, chemosis of the ocular conjunctiva occurs, the tissue rising up, and overlapping the edge of the cornea sometimes to such an extent as to quite obscure it. Meanwhile, the cornea may become infiltrated, more especially near its margin, and an ulcer may form. This is apt to progress in a crescentic course, the gonococci getting between the layers of the cornea rapidly breaking down the tissue, and it often leads to rapid perforation, with possible loss of the contents of the globe, the lens and vitreous body may come away, or only a protrusion or hernia of the iris take place. This may be returned, but not cut off. About the fourth or fifth day from the first appearance of the symptoms the inflammation reaches its acme, and a profuse purulent discharge commences to escape. The height of inflammation with exacerbations lasts from 24 to about 72 hours, and then the tenseness of the lids subsides gradually, the discharge thins, the chemosis lessens, and the tedious stage of convalescence of the eye begins. This may last several weeks, and it often terminates in a thickened and roughened condition of the palpebral conjunctiva, or the tissue may return to various degrees approaching the normal.

If the cornea has sloughed there will be opacity, with bulging at that point, anterior staphyloma; if severe ulceration without perforation, a deep opacity; if perforation with loss of the contents of the globe, a shrinking of the eyeball, or the eyeball may be destroyed by the gonococci reaching the interior of the globe and setting up panophthalmitis; if the cornea escape there will be recovery to useful vision.

As soon as the patient is seen he should be isolated, the sound eye should be carefully washed out, and a solution of nitrate of silver one-half per cent. or a 5 per cent. solution of protargol in distilled water; then this eye should be carefully bandaged, with an antiseptic pad under the bandage; or, better still, a Buller's shield, which will enable him to use the eye to look about, applied. This is made by taking a concavo-convex watch-glass and placing it between two layers of Bristol strapping, the outer one of which overlaps the inner; having first cut holes in them rather smaller than the glass.

This apparatus is applied carefully along the supercilliary ridge, and down the side of the nose, and sealed where it meets these parts with flexible collodion, or Friar's Balsam. The diseased eye is then inspected as carefully as possible, the discharge washed away with lotion of perchloride of mercury, 1 in 4,000, used carefully in a small ʒii. glass syringe, being very careful not to touch the eye, especially the cornea, with the nozzle.

Then a solution of nitrate of silver, 20 grs. to the ounce, should be wiped all over the surface of the conjunctival sac, or if that be impossible, dropped in with an eye-dropper, and two or three minutes afterwards washed out with a common salt lotion. The eye should be attended to night and day hourly, even half-hourly in severe cases, the discharge mopped away, and a few drops of the solution of perchloride of mercury as above used. A saturated solution of boracic acid, used warm, will do for the washing out, and instead of the perchloride solution, a 5 or 10 per cent. solution of protargol may be used, the nitrate of silver, reduced to 10 grains to the ounce, may be used again when the reaction of the first application has passed, that is in about eight or ten hours, and repeated as long and as strong as necessary, the strength being weakened as the disease is yielding.

When the cornea is pressed on by the chemosed conjunctiva, which is hard from fibrino.plastic deposit, it runs a risk of being strangulated, and of necrosing. It is then often advisable to snip the swollen conjunctiva with scissors, or even to remove a piece of the conjunctiva, or the whole ring surrounding the cornea. The congestion may be reduced and the pain much relieved by the application of two or three leeches to a spot near the outer canthus. The patient's temperature often is elevated and is best treated with aconite, liq. ammoniæ acetatis, and spts. etheris nitrosi, or in some cases with quinine.

THE SHIELD IN SITU.—Right Eye. *o*, Outer layer of plaster; *v*, Open corner for ventilation.

*o*, Outer layer of plaster seen from within sticky side; *i*, Inner layer of plaster; *g*, Watch glass between layers.

DIAGRAMMATIC SKETCH OF BULLER'S SHIELD.

The urethritis must be treated in the usual way, a half per cent. solution of protargol being injected, or the same drug used in bougies made with ol. theobroma, and a capsule of sandalwood oil given every fourth or fifth hour. The patient will suffer from want of sleep, owing to the necessity of washing out the eye so frequently; he should, therefore, be allowed to doze as much as possible, and a soothing draught, such as we have in a teaspoonful of bromidia, may be given at night.

The attendants must carefully wash their hands after each dressing, and avoid touching the sound eye or allowing the soiled dressings to pass over to the side of the sound eye. When the watch-glass or the sound eye want moistening or drying, a piece of absorbent wool rolled on the end of a wooden match is a useful instrument.

Despite all the treatment, care, and attention employed in the conduct of such cases the results will too often be very disappointing.

## Figures and Tables

**Figure f1:**